# The Poor Laws

**Published:** 1899-08-26

**Authors:** 


### The Hospital

A Journal of

The Medical Sciences and Hospital Administration.

Vol. XXVI.—No. 674.] Edited by Sir Henry Burdett, K.C.B., and Solomon C. Smith, M.D., M.R.C.P. [August 26, 1899.

### The Poor Laws.

If there be any class of the community to which the Divine assertion, "the poor ye have always with you," is more applicable than to others, that class is undoubtedly composed of the members of the medical profession. With many others philanthropy is at best an occasional recreation, and sometimes not an unprofitable one; with the doctor it takes its place among the daily duties of his calling. His visits, like those of death, are paid as much to the hovels of paupers as to the palaces of kings; nay, much more in proportion, as the paupers are the more numerous. In great cities it may often be the case that the poor are chiefly attended in sickness, so far as their own homes are concerned, by doctors who are comparatively seldom consulted by persons of a higher class; and the medical relief given in hospitals is not generally conducive to the establishment of any other relations than those which exist in the out-patient room or the ward. But in provincial towns not of the first magnitude, and in country districts generally, the doctor of the peer will also be the doctor of the peasant, and, even more than the clergyman, will be the means of making known the wants of the poor to those who are able and willing to supply them. He is a clear-sighted, but seldom a harsh, critic, whose sympathy with genuine misfortune is never failing, while it is seldom entirely withheld even from those whose misfortunes are largely created by their faults. He sees the poor as they are, their virtues as well as their vices, the former without affectation, the latter without concealment. They know that he is not to be humbugged, and they refrain, as a rule, from the attempt. On the whole, and as a net result, the doctor has, we believe, a genuine liking for a large proportion of the poor, and in many instances no small degree of respect for them. The conditions which strip bare their minds to him are equally operative among the rich; and the removal of disguises furnished by convention or by policy permits comparisons which are not always entirely to the advantage of the latter. There are few doctors who have not seen among the poor many examples of true heroism; there are few who have not seen among the rich many examples of shivering and miserable cowardice. Neither one class nor the other can claim any monopoly of courage or any monopoly of virtue; and the pictures of manual labour drawn by such writers as Day and Cobbett are only a thought less absurd than those drawn of "Guardsmen" by such writers as Ouida or a few other contemporary novelists. In England, fortunately for us, we have no distinction of classes. The descendants of the peer may sink into the proletariat; the descendants of the peasant may rise to the peerage. The inherited results of education and of authority, the inherited results of ignorance and of submission, are alike widely distributed among our people.

A "reform," or at least an alteration, of the existing Poor Law is said by professional politicians to be "in the air," and is almost certain to be brought forward in an early session of Parliament, as a means of creating political capital for its promoters. It has long been a source of grievance among doctors that they, as a profession, have little or no political influence. Now, the only abiding source of political influence is knowledge. "Cries" and claptrap are like quackery, they have their little day and are forgotten. Knowledge is like legitimate medicine, it not only endures, but grows and increases by exercise. The actual state of the poor, their real needs, and the probable operation of any measures professedly directed towards the supply of these needs are far more within the cognisance of doctors than of any other class of the community; and it follows that proposals for legislation about the poor would afford to doctors, if they chose to embrace them, unrivalled opportunities for the display of political capacity, and for the assertion and establishment of a position, in regard to a great public question, which they have never hitherto assumed, and which, if they did assume it, could not fail to react greatly to their advantage in many other directions. It is certain that such proposals, whatever they may be, will at least require an increase of rates, and that, on this ground alone, they will be strenuously opposed, without the least reference to their being wise or foolish, calculated to conduce to the national prosperity, or only to increase the national burdens. The United Kingdom contains a large number of local medical societies, which meet at stated periods, and proceed to dinner after having discussed Dr. A.'s case of multiple sarcoma, or Mr. B.'s successful performance of some new or serious operation. Why should not these societies apply themselves to a careful and statesmanlike consideration of the problems arising out of the existence and the necessities of the poor? Why should they not prepare themselves to furnish sound arguments to their representatives in the House of Commons, to support a judicious measure, or to oppose an injudicious one, by memorials containing careful reasoning based upon unassailable information? If they would do this they would not only immeasurably raise the profession in the estimation of the public, but they would also pave the way for the favourable consideration of reforms by which the profession itself would be more immediately affected.

